# *Begoniayenyeniae* (Begoniaceae), a new species from Endau Rompin National Park, Johor, Malaysia

**DOI:** 10.3897/phytokeys.110.25846

**Published:** 2018-11-01

**Authors:** Joanne Pei-Chih Tan, Sheh May Tam, Ruth Kiew

**Affiliations:** 1 Forest Research Institute Malaysia, 52109 Kepong, Selangor, Malaysia Forest Research Institute Malaysia Kepong Malaysia; 2 School of Biosciences, Taylor’s University, Jalan Taylors, 47500 Subang Jaya, Selangor Darul Ehsan, Malaysia Taylor’s University Subang Jaya Malaysia

**Keywords:** new species, *
Begonia yenyeniae
*, *
Begonia rajah
*, *
Begonia reginula
*, ornamental, conservation

## Abstract

*Begoniayenyeniae* is a new species of horticultural value known only from the Endau Rompin National Park, Peninsular Malaysia. It is similar to *Begoniarajah* with which it had previously been confused in the number of tepals and leaf characters. The new species is compared with three similar species, *B.foxworthyi, B.rajah* and *B.reginula* and photographs of all four species and descriptions of *B.yenyeniae* and *B.rajah* are provided. Molecular analysis using the *ndhF-rpl132* chloroplast marker confirms the four species as distinct. Amongst native species, the three variegated species, *B.yenyeniae, B.rajah* and *B.reginula*, are some of the most popular Malaysian begonias in cultivation. Based on its restricted distribution, *Begoniayenyeniae*, under the IUCN Red List Categories and Criteria, is assessed as Critically Endangered.

## Introduction

Asian begonias are prized for the eye-catching variety of colours and patterns of their variegated leaves and so are targets for horticultural interest as a genetic source for new hybrid cultivar development. However, a vast array of begonias is of conservation importance either because they have restricted distributions, being known from very few localities or they are confined to a specific habitat niche. For example, of the 54 native taxa in Peninsular Malaysia, 57% are threatened with 24 taxa assessed as Critically Endangered and 21 species are known from a single locality (Chua et al. 2009). The great majority grows in primary forest, frequently on rocks beside streams or waterfalls, where they are threatened by loss of habitat. Forest cover declined rapidly from 70% in 1960 to 41% in 2000 in Peninsular Malaysia due to massive land development schemes (Chua 2010), disturbance from eco-tourism activities and for pretty species, collecting from wild populations for cultivation or sale. At least one begonia species is extinct (Kiew 1989a) and several have not been found since their original discovery. Ironically, some species, that may be extinct in the wild, live on because they are popular with plant enthusiasts and are available from commercial growers.

As pristine forest diminishes in the face of extraction of timber or land clearance for the planting of oil palm and other crops and for infrastructure development, forests are becoming valued for the ecological services they provide and as a source of medicinal and ornamental plants or minor forest products, as well as for tourism and recreation. However, the commercial value of native species is often realised outside the country of origin so minimal benefit is generated locally. In Malaysia, the introduction of wild native species into horticulture has been haphazard. In recognising this, the Forest Research Institute Malaysia (FRIM) started a plant domestication project to trial attractive wild species for their horticultural potential with the aims of not only enabling Malaysia to benefit commercially from its rich biodiversity (Tan 2009), but also to illustrate that other species, besides timber trees, are of commercial value and that their habitat should also be protected. In addition, making attractive species readily available would help to take pressure off collecting, often illegally, from wild populations.

This new species was one of the candidates of this project. It was discovered in 2002 by Dr Sam Yen-Yen in the Endau-Rompin National Park in the southern state of Johor. It proved to be amenable to cultivation and, unlike many native begonias that need constant high humidity, it does not need to be grown in a covered terrarium. So the species was ‘bulked up’ and offered to the horticulture trade to test its potential. Unfortunately, the local horticultural market was reluctant to invest in the marketing of this species on a commercial scale but, based on information available on social media and websites, it spread widely amongst specialist growers. Individual plants from the original Johor collection continued to be grown for further studies in the Kepong Herbarium (KEP) nursery. The Johor plants have been identified as *Begoniarajah* Ridl. because they have the same habit, number of tepals (four in the male flower and three in the female) and similar attractive leaf colour and surface (Kiew 2005). Comparison between these plants and those of *B.rajah*, obtained commercially, showed important differences and indicated that they were different taxa. To test this supposition, molecular phylogenetic analysis of the new species, *B.rajah* and *B.reginula*, was performed.

*Begoniarajah* is one of the most decorative Malaysian begonias due to its striking and uniquely contrasting bronzy-green leaf colour. It was discovered in 1892 and gained fame in 1894 when it was sent to England and won a First Class Certificate from the Royal Horticultural Society (Anonymous 1894a). After more than a century, it is remains popular in cultivation today (Thompson 1976, Kiew 1989b, Tebbitt 2005). However, its origin is still shrouded in mystery. Ridley (1892) recorded only that it was collected by a native collector from Tringganu (the Malaysian state of Terengganu). Despite continuous botanical exploration, it has never been re-found, although another species, *Begoniareginula* Kiew (Figure 4), similar in leaf characters, was discovered in Terengganu, but it proved to be a distinct species based on the different number of tepals (Kiew 2005, Tan 2016).

With recent publications of higher level (sectional) molecular phylogenies of *Begonia* (Moonlight et al. 2018, Tebbitt et al. 2006, Rajbhandary et al. 2011), especially involving species from South East Asia (Thomas et al. 2011, 2012), it is now timely to perform a molecular phylogenetic study that includes greater representation of *Begonia* species from Peninsular Malaysia.

## Methods

**Figure 1.**
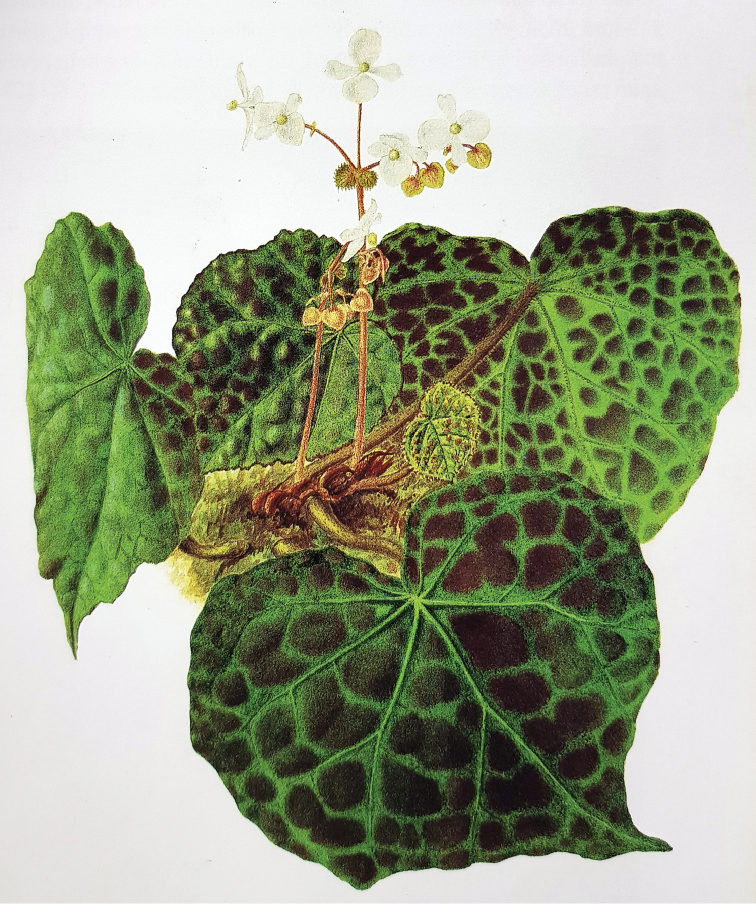
Watercolour painting of *Begoniarajah* of an original wild-collected plant grown in the Botanic Gardens, Singapore. (Reproduced with permission of the Singapore Botanic Gardens)

The description and measurements of the new species are based on herbarium specimens deposited in the Kepong Herbarium, Forest Research Institute Malaysia (KEP) and Singapore Botanic Gardens Herbarium (SING) and fresh material cultivated in the KEP nursery. The diagnostic characters of the new species were compared with those of the type specimen of *B.rajah* in the Herbarium of Royal Botanic Gardens, Kew (K), the watercolour painting made from the original *B.rajah* plant (Figure 1) and early botanical descriptions. As *B.rajah* was confused with the new species (Kiew 2005), a new description is provided here. Other similar species, *B.reginula* Kiew and *B.foxworthyi* Burkill ex Ridl. are shown by molecular data to share a close relationship and are compared with the new species based on herbarium specimens in KEP, protologues (Kiew 2005) and living material available in the KEP nursery. The conservation status of the new species is assessed following the standard IUCN Categories & Criteria (IUCN 2012).

### Species relationships amongst *B.yenyeniae*, *B.foxworthyi*, *B.rajah* and *B.reginula*

Interspecific relationships amongst the proposed new species (*B.yenyeniae*), *B.foxworthyi*, *B.rajah* and *B.reginula* were inferred from our current, on-going phylogenetic study that focuses on *Begonia* species from Peninsular Malaysia. DNA sequences for the chloroplast *ndh*F-*rpl*32 intergenic spacer region were generated for 48 taxa of *Begonia* which included species mostly from Peninsular Malaysia (Suppl. material 1).

### DNA Extraction, PCR amplification and DNA sequencing

DNA of *Begonia* species was extracted from living material or silica gel dried material using the NucleoSpin Plant II kit following the manufacturer’s protocols (Macherey Nagel, Germany). Amplification of the chloroplast *ndh*F-*rpl*32 intergenic spacer was done using the forward and reverse primers ndhFBeg-F and trnLBeg-R, respectively, designed by Thomas et al. (2011). Each PCR reaction contained 12.50 µl of 2X GoTaq Green Master Mix (Promega, U.S.A.), 1 µl of each forward and reverse primer (10 µM) (Integrated DNA Technologies, U.S.A.), 1 µl of bovine serum albumin (New England BioLabs Inc., U.S.A.), 7.50 µl of RNase free water (Bio Basic Inc., Canada) and 2 µl (~ 100 ng) of DNA template to make up 25 µl total volume. The following PCR profile was used: initial denaturation at 95 °C for 5 min, followed by 40 cycles of denaturation at 95 °C for 1 min, primer annealing at 55 °C for 1 min and extension at 72 °C for 2 min; followed by a final extension step at 72 °C for 5 min. Amplicons were visualised using 1.0% agarose gel electrophoresis stained with SYBR Safe DNA Gel Stain (Invitrogen, U.S.A.). Samples with the expected size (~ 750 bp) were purified using the Wizard SV Gel and PCR Clean-Up Kit (Promega, USA) before being sent for DNA sequencing using both the forward and reverse primers by First BASE Laboratories Sdn Bhd (Selangor, Malaysia). Both the forward and reverse sequences were manually edited (cleaned) and aligned to generate a consensus sequence for each sample using the programme BioEdit. Multiple sequence alignment of the generated sequences together with the *ndh*F-*rpl*32 sequences of 79 Asian *Begonia* species (from an earlier phylogenetic study by Thomas et al. 2011) downloaded from GenBank was performed using Clustal Omega (http://www.ebi.ac.uk/Tools/msa/clustalo/) using default parameters. The resulting alignment file was then manually edited before analysis and is available from the corresponding author upon request.

### Data analysis

The final alignment file, comprising of 126 taxa and 1244 characters, was subjected to Bayesian analysis performed using MrBayes 3.2.6 on XSEDE via the CIPRES portal (https://www.phylo.org/). Following Thomas et al. (2011), the GTR + G model was selected for the analysis with the following MCMC parameters: number of generations = 1,000,000; number of runs = 2; number of chains = 4; temperature parameter = 0.20; sample frequency = 1000; and burn-in fraction = 0.25. The resulting 50% majority rule consensus tree with node support expressed as posterior probabilities was viewed using FigTree (http://tree.bio.ed.ac.uk/software/figtree/). The programme MEGA 6 (Tamura et al. 2013) was used to compute values of genetic distance (gd) using the Kimura 2 parameter (K2P) model for interspecific comparisons between the three species, *B.yenyeniae*, *B.rajah* and *B.reginula*.

## Results

Results of our on-going study from Bayesian analysis of 47 taxa of *Begonia* from Peninsular Malaysia, which included accessions of *B.rajah, B.reginula* and the new species *B.yenyeniae*, together with 79 other Asian species using the *ndh*F-*rpl*32 chloroplast marker, showed that all three species resolved together in a strongly supported polytomous clade (Bayesian posterior probability (BPP) support = 1) where *B.yenyeniae* clearly separated in its own subclade (BPP support = 0.98) with accessions of *B.foxworthyi* and *B.nurii*. The tree shows that the *B.yenyeniae* accession resolved in a relatively long branch which lends support to its separation as a proposed new species. Part of the tree highlighting relationships amongst *B.rajah*, *B.reginula*, *B.foxworthyi* and *B.yenyeniae* are presented in Figure 5. Values of genetic distance (gd) computed under the Kimura 2 parameter (K2P) model for interspecific comparisons amongst the three species showed the gd between *B.yenyeniae* and *B.rajah* as 0.024; *B.yenyeniae* and *B.reginula* of 0.021 and *B.rajah* and *B.reginula* of 0.01. The two *B.foxworthyi* samples (from Bentong and Merapoh, respectively, that resolved in the same subclade as *B.yenyeniae*) recorded the same gd values from *B.reginula* at 0.006; whilst the gd between the two *B.foxworthyi* samples and *B.yenyeniae* are 0.015. The gd values between *B.foxworthyi* (Bentong) and *B.foxworthyi* (Merapoh) from *B.rajah* are 0.01 and 0.012, respectively.

The *ndh*F-*rpl*32 DNA sequences of *B.yenyeniae*, *B.rajah* and *B.reginula* have been deposited to GenBank (accession numbers MH454102, MH454103 and MH454104, respectively). Thus, molecular data support the close relationships of these species and the genetic distinction of *B.yenyeniae*. It is noted that the phylogenetic tree shows that a single chloroplast marker has limited power to resolve interspecific relationships in this group and, clearly, additional molecular markers are required as certain accessions of the same species did not resolve in clearly differentiated clades.

### Taxonomy

#### Begonia yenyeniae

Taxon classificationPlantaeCucurbitalesBegoniaceae

J.P.C.Tan sp. nov.

urn:lsid:ipni.org:names:77191563-1

Figure 2

##### Section.

*Jackia* M.Hughes

##### Diagnosis.

Similar to *Begoniarajah* Ridl. (1894:213) in its handsome leaves, striking brownish-pink to brownish-red with greenish-yellow veins in young leaves becoming bronzy variegation at maturity; in its creeping growth habit, number of tepals in male (4 tepals) and female flowers (3), palmate leaf venation and many-flowered cymes, but several notable characters distinguish the new species, including its orbicular-reniform leaf blades (vs. subrotund with an abruptly acute apex in *B.rajah*), smaller stipules, 9–12 × 3–4 mm, three times longer than wide (vs. 15–20 × 10–12 mm, less than twice as long as wide), smaller ovate or obovate bracts 2–3 × 1.5–2 mm (vs. bracts bowl-shaped, wide ovate, 5–8 × 7.5–8 mm), margin shallowly crenate (vs. margin angular), bullate leaf surface (vs. conspicuously pronounced bullate). Furthermore, the leaf colour of cultivated *B.rajah* is more vivid and, in contrast, its tepals, stipules and bracts are also a deeper shade of pink, compared to those of *B.yenyeniae*.

It is also similar to *B.reginula*Kiew (2005: 218) in its habit, leaf colour, less pronounced bullate blade, palmate venation, bracts, ovary with 3 equal wings of similar shape, but *B.yenyeniae* is different in its relatively smaller and narrower tepals 5–7 × 6 mm (vs. 6–10 × 9–11 mm in *B.reginula*), 3 in male or 4 tepals in female flowers (vs. 2 tepals in both male and female flowers) and rounded base (vs. subcordate), stipules with prominently keeled (vs. keel absent), and apex rounded (vs. apex attenuate).

Although molecular data indicate a close relationship to *B.foxworthyi* ((1925: 311), the latter species is morphologically distinct in its conspicuously oblique leaf with an acute to acuminate apex (vs. orbicular-reniform with a rounded apex), its entire margin (vs. crenate) and plain green, non-bullate leaf surface (vs. purplish-green to brownish-purple and bullate) and its male flowers with 2 tepals (vs. 4 tepals). In addition, it usually grows on limestone substrates (vs. confined to granite substrates.

##### Type.

Peninsular Malaysia. Johor, Mersing, Endau-Rompin National Park, Sungai Selai, 14 April 2018, Kiew FRI 81950 (holotype E!; iso-: K!, SAR!, SING!).

##### Description.

Lithophytic herb with rhizomatous stem. Indumentum of soft hairs, usually cream- coloured, sparse on stipules (mostly on the keel and 0.5–1.5 mm long), petioles more densely hairy towards blade (2–3 mm), leaf margin (hairs ca. 1 mm) and veins beneath (ca. 1 mm) but sparse (ca. 0.5 mm) or absent on ventral surface of outer tepals and on peduncle hairs hardly visible to the naked eye. **Stems** creeping, apex usually slightly erect, branched, 5–9 mm thick, light yellowish-green; stipules 3 per node, two triangular, abruptly narrowed to an attenuate apex terminating in a hair, keeled from base to apex, margin entire and translucent, 9–12 × 3–4 mm, pale yellowish-green, one narrowly lanceolate, ca. 3 mm long, persistent. **Leaves** tufted, alternate, 2–3 mm apart; petioles terete, 2.5–3.5 mm across, up to 12.5 cm long, pale brownish-pink to darker pink towards blade; blades thinly succulent, glabrous, orbicular-reniform, asymmetric, 8–11.5 × 9.5–13.5 cm, scarcely angular, margin somewhat crenate with acute teeth bent abruptly downwards between teeth, ciliate, basal lobes cordate, overlapping when mature, moderately raised between veins, light purplish-green to dull brownish-purple, young blades brownish-pink to brownish-red, paler beneath; veins palmate, slightly prominent towards the base but impressed where branched towards the margin, prominent beneath, lateral veins ca. 2–3 pairs, greenish-yellow when young and whitish-green when mature. **Inflorescences** axillary, more or less erect, 10–23 cm long, brownish-red, peduncles 8–18.5 cm long, two main branches 2–3 cm long, pedicels 6–9 mm; bracts in pairs at node of peduncle, glabrous, elliptic-ovate or obovate, margin towards apex laciniate, 2–3 × 1.5–2 mm, light yellowish-green sometimes with a faint tinge of pink, persistent. **Male flowers** with 4 tepals, margin entire, 1–1.2 × 1.1–1.4 cm; outer 2 tepals rotund, concave at centre, 5–7 × 6 mm, pale greenish-white or light pink or pale pinkish-white, inner 2 tepals obovate, apex rounded or sometimes retuse and impressed along centre, ca. 5 × 3 mm, white; stamens numerous, torus ca. 1 mm long, stamen mass globose, symmetric, ca. 2.5 mm across; anthers obovoid-oblong, tip emarginate, ca. 0.5 mm long, dehiscing through 2 longitudinal slits. **Female flowers** with a light yellowish-green ovary sometimes with faint light pink tinge, ca. 5.5 × 9 mm, locules 3, wings 3 equal, placentation axile, 1 placenta per locule, each placenta usually with 2 minute branches at the base; usually tepals 3, outer 2 tepals, rotund, concave at centre, ca. 4 × 4.5 mm, white with faint green tinge; styles and stigmas 3, 1.5–2 mm, style light greenish-yellow, stigma pale yellow, papillose, spiral band. **Capsules** ca. 6 ×12 mm, locules 3, splitting longitudinally between locules, wing 3 equal, ca. 4 mm wide, surface glabrous, styles and stigmas persisting after tepals have fallen, dangling on a fine, thread-like pedicel ca. 5.5 mm long. **Seeds** numerous, barrel-shaped, 0.3–0.34 × 0.2–0.22 mm, collar cells slightly more than half the seed length, surface sculptured.

##### Distribution.

Endemic in Peninsular Malaysia, Johor, Mersing District, Endau-Rompin National Park, Sungai Selai. It is apparently a rare species as it is known only from the type locality in Endau Rompin National Park.

##### Etymology.

Named after Dr Sam Yen-Yen, Malaysian botanist, specialist in Zingiberaceae who first discovered the species and recognised its potential as an ornamental plant.

##### Conservation status.

Critically Endangered CR B2ab(iii, v), D1. Although the type locality is within a Totally Protected Area, it is known from only one population about 1.5 km^2^ and its habitat is threatened by ecotourism activity and illegal collecting. The area of observed population covers about 1.5 km^2^ (P.T. Ong pers. comm.).

##### Ecology.

In primary lowland mixed dipterocarp forest, growing on moss-covered rocks, rarely epiphytic, near a waterfall in deep shade.

##### Note.

**Figure 2.**
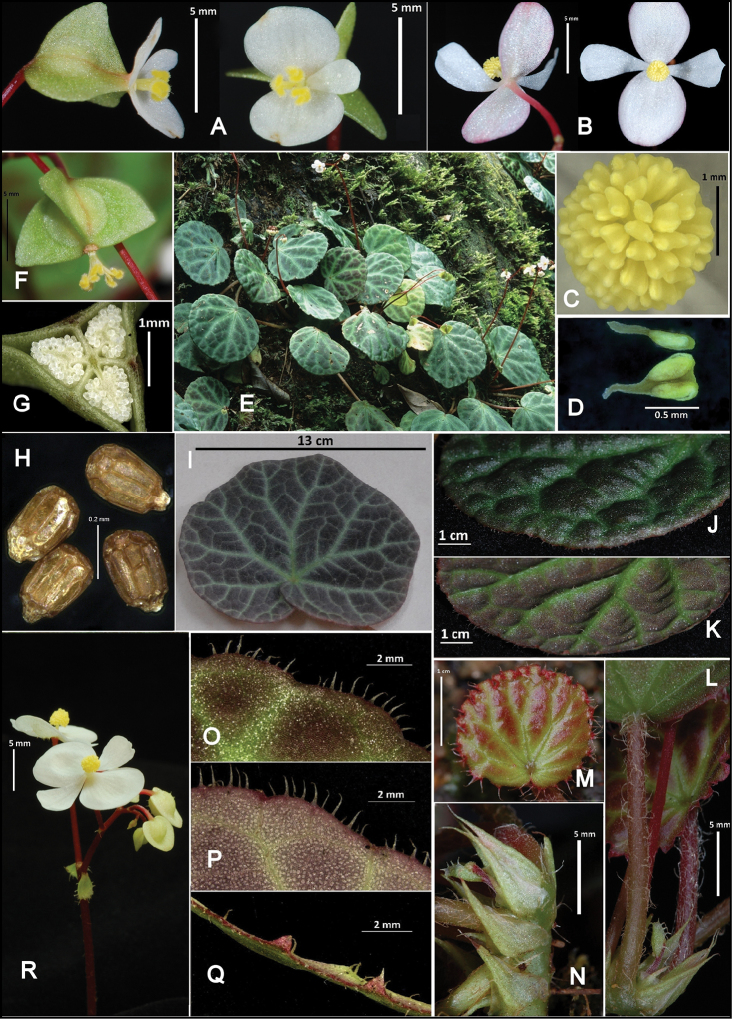
*Begoniayenyeniae* J.P.C.Tan, sp. nov. **A** Side and front view female flower **B** Back and front view of male flower **C** Stamen mass **D** Anthers **E** Habitat: moss-covered rocky slope by waterfall **F** Young fruit with stigma still attached **G** Transverse section of fruit **H** Seeds **I** Mature leaf **J** Upper leaf surface (moderately bullate) **K** Veins completely prominent on lower leaf surface **L** Petiole **M** Young blade **N** Stipules **O–Q** Upper, lower and side view of leaf margin **R** A pair of bracts and bracteole at peduncle and rachis; hairs scarcely on ventral surface of outer tepals. (Photographs by **E** Y.Y. Sam, **D** P.T. Ong)

The ovary of species in section Jackia have three locules, each with an unbranched placenta, but in this species two vestigial branches are present near the base of the placenta (Figure 2G). This is also seen, but is less pronounced, in *B.rajah*.

##### Other specimens examined.

**Peninsular Malaysia.** Johor: Mersing, Endau-Rompin National Park, Sungai Selai, 15 Aug 2002, Sam et al. FRI 47082 (KEP!), 24 July 2012, Hairul et al. FRI 78570 (KEP!, SING!).

#### Begonia rajah

Taxon classificationPlantaeCucurbitalesBegoniaceae

Ridl. ex Rolfe

Figure 1 , 3

Gardeners’ Chronicle XVI (1894) 213, nom. nud.; Rolfe, Bulletin of Miscellaneous Information (1914) 327; Irmscher, Mitteilungen aus dem Institut für allgemeine Botanik in Hamburg 8 (1929) 96; Tebbitt, Begonias: Cultivation, Identification, and Natural History (2005) 198; Kiew, Begonias of Peninsular Malaysia (2005) 216, pro parte.

##### Section.

*Jackia* M.Hughes

##### Type.

Peninsular Malaysia. Terengganu, 1892, *Native collector s.n.* (holotype SING ex K!). **Description.** Dwarf herb with rhizomatous stem. Indumentum of soft hairs, usually pale pink, dense on stipule (1.5–2 mm long), petiole (cream-coloured, 2–4 mm), leaf margin (1–1.5 mm) and veins beneath (2–2.5 mm), peduncle (cream-coloured, 2–2.5 mm). **Stems** creeping, ca. 9 mm thick, light yellowish-green; stipules lanceolate-oblong or triangular, 15–20 × 6–10 mm, apex attenuate terminating in a hair, keeled from base to apex, margin entire and opaque, brownish-pink to scarlet, persistent. **Leaves** tufted, alternate, ca. 5 mm apart; petioles suberect, terete, ca. 5 mm across, 8–25 cm long, light yellowish-pink; blades rigid, succulent, glabrous, broadly ovate with an abruptly acute apex or radical oblique cordate, 7–15 × 6–15 cm, prominently bullate, margin ciliate, angular with abruptly acute teeth, basal lobes cordate, slightly overlapping when mature, notably dark brownish-purple blotches, polished shining surface, young blades brownish-pink to vivid brownish-red, paler beneath; veins palmate, prominent towards the base and slightly impressed near margin, prominent beneath, lateral veins ca. 1–3 pairs, greenish-yellow when young, yellowish-green when mature. **Inflorescences** axillary, almost erect, many-flowered cyme, 20–25 cm long, peduncles 7–12 cm long, brownish-pink, two main branches ca. 1.3 cm long, pedicels slender, ca. 1 cm; bracts in pairs at node of peduncle, glabrous, broadly ovate, bowl-shaped, subacute, margin laciniate 5–8 × 7.5–8 mm, light yellowish-pink, persistent; bracteole narrowly ovate, 2–3 mm long, pale yellowish-pink. **Male flowers** with 4 tepals, margin entire, ca. 1.8 cm across, rose pink; outer tepals 2, obovate-orbicular or elliptic-ovate, 6–8 × 5–8 mm, inner 2 tepals obovate or narrow oblong, apex rounded or sometimes retuse and impressed along centre, ca. 5 × 3 mm; stamen mass globose, symmetric, 3 × 2.5 mm; anthers broadly obovoid-oblong, tip rounded, ca. 0.7 mm long, dehiscing through 2 longitudinal slits. **Female flowers** with a pale pink ovary with darker pinkish margin, 7–8 mm, locules 3, placentation axile, 1 placenta per locule, each placenta usually with 2 minute branches at the base, usually 3 tepals rarely 2, outer 2 tepals widely elliptic-ovate, concave at centre, ca. 6–7 mm, pale pink; styles and stigmas 3, 2–2.5 mm, style light greenish-yellow, stigma pale yellow, papillose, spiral band. Capsules ca. 1.2 cm across, locules 3, splitting longitudinally between locules, surface glabrous, dangling on a fine, thread-like pedicel 1 cm long. **Seeds** not seen by us.

**Figure 3.**
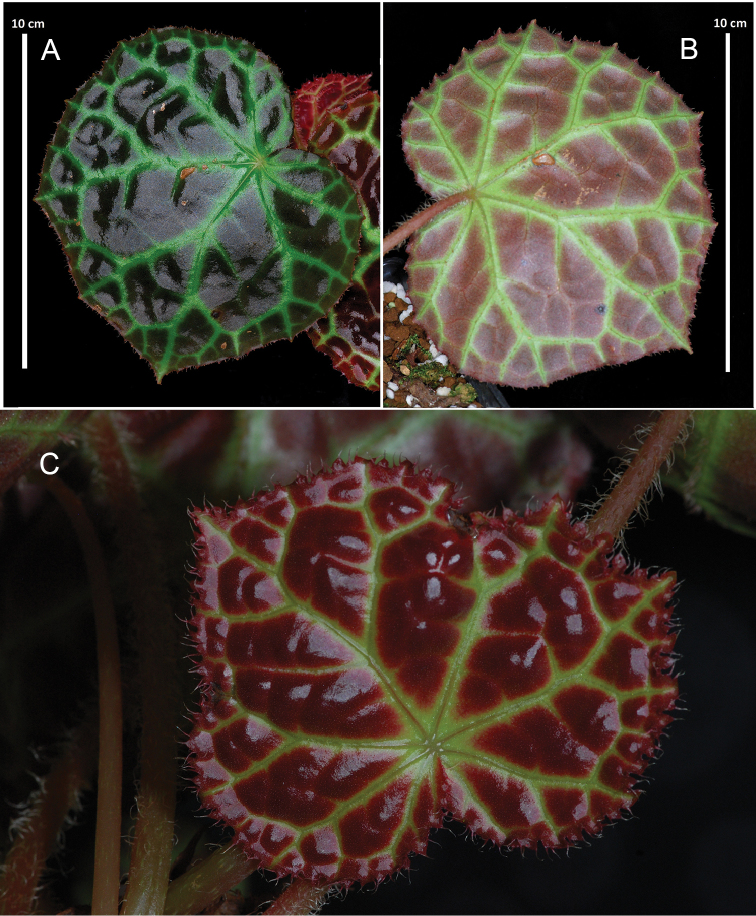
*Begoniarajah* Ridley (cultivated) **A** Upper leaf surface **B** under surface **C** Young blade.

**Figure 4.**
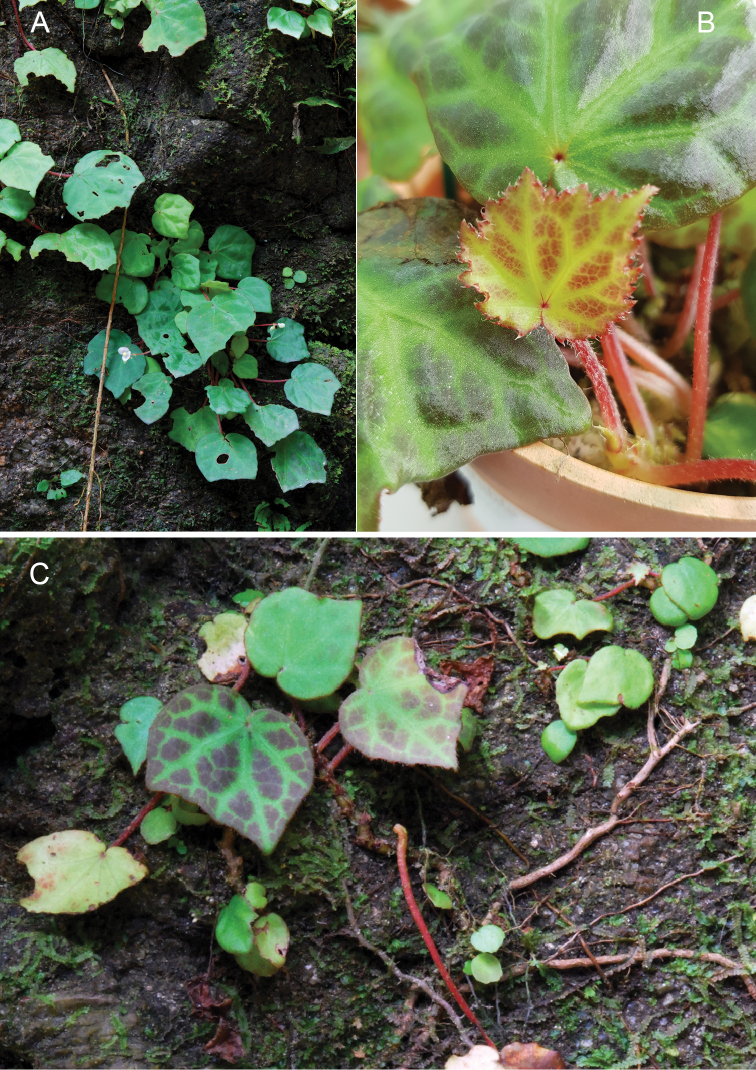
*Begoniareginula* Kiew **A***In situ*: clinging to moss-covered granite boulders **B** Young blade **C** Mature leaf of coloured form.

##### Distribution.

Endemic in Peninsular Malaysia, Terengganu (without specific locality). Apparently restricted in its distribution because, despite continuous botanical collecting in Terengganu and elsewhere in Peninsular Malaysia, it has not been re-found since it was first collected in 1892 (Kiew, 2005).

**Figure 5.**
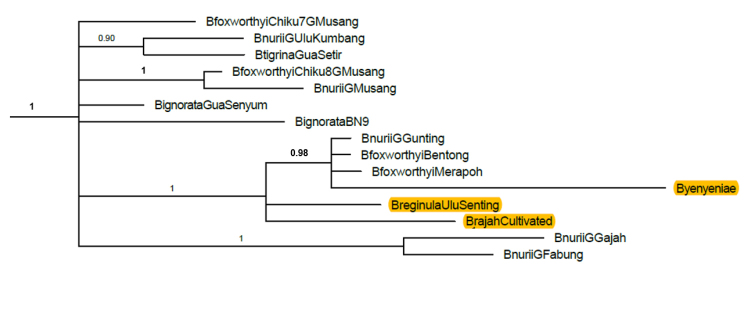
Part of the Bayesian 50% majority rule consensus tree from the analysis of chloroplast *ndhF*-*rpl32* region depicting relationships amongst *B.foxworthyi*, *B.rajah*, *B.reginula* and *B.yenyeniae*. The locality is indicated at the end of the species name for species with multiple samples. Numbers on the branches indicate Bayesian posterior probability (BPP) support values.

**Table 1.** *Begoniarajah*, *B.yenyeniae*, *B.reginula* and *B.foxworthyi* compared.

Character	* Begonia rajah *	*Begoniayenyeniae* sp. nov.	* Begonia reginula *	* Begonia foxworthyi *
Leaf: texture	thickly succulent polished shining prominent bullate	thinly succulent	thinly succulent slightly raised in between veins	thinly succulent
Surface		moderately raised in between veins		glossy, veins slightly impressed
Leaf shape	subrotund	orbicular-reniform	broadly ovate	almost rotund apex short- pointed
	apex abruptly acute	apex rounded	apex abruptly attenuate angular	not angular
	angular	scarcely angular		
Tepal no. in male flower	4	4	2	2
Tepal no. in female flower	3	3	2	2
Outer tepal size (mm)	6–8 × 5–8	5–7 × 6	6–10 × 9–11	4–6 × 4–6
Outer tepal: shape	widely ovate or cordate	rotund	widely ovate or rotund	broadly ovate to rotund rounded
apex	obtuse	rounded	rounded or acute subcordate	subcordate
base	subcordate	rounded		
Stipule: shape	lanceolate-oblong or triangular	narrowly triangular,	narrowly triangular,	narrowly triangular
size (mm)	15–20 × 10–12	9–12 × 3–4	5–7 × 2–3	7–12 × 2–5
colour	Brownish-pink to scarlet	light yellowish-green sometimes with a faint pink tinge	pale greenish yellow or reddish yellow	red
Keel on stipules	from base to apex	from base to apex	absent	from base to apex
Bract: shape	broadly ovate, bowl-shaped	elliptic-ovate or obovate, curved or flat	obovate, curved or flat	obovate, flat
size (mm)	5–8 × 7.5–8	2–3 × 1.5–2	3–4 × 1–3	4–6 × 2–4
Hair density:				
petioles	hairy	sparsely hairy, more hairy near leaf base	sparsely hairy	densely hairy
stipules	densely hairy	sparsely hairy	hairy	densely hairy
peduncles	hairy	sparsely covered, hairs inconspicuous	without hairs	sparsely hairy
Ovary colour	pink or pale pink	light yellowish-green	light yellowish-green	reddish or dark pink

##### Etymology.

From a Sanskrit word *raja* = king, presumably referring to it as the most beautiful begonia species.

##### Conservation status.

Extinct in the Wild (EW). However, it survives in cultivation since its first introduction into cultivation in 1894 (Anonymous 1894b, 1895).

##### Ecology.

Its natural habitat is not known but judging from its growth requirements in cultivation, it probably grew in cool and shaded conditions. **Other specimens examined. Peninsular Malaysia.** Herbarium specimens made from cultivated plants in Royal Botanic Gardens, Kew Anonymous s.n. 1903 (K!); Sander F. et al., s.n. 1913 (K!); Anonymous s.n. August 1913 (K!).

## Supplementary Material

XML Treatment for Begonia yenyeniae

XML Treatment for Begonia rajah

## Supplementary materials

Supplementary material 1 List of taxa with accession and location information for the 48 samples of Begonia included in the Bayesian analysis using ndhF-rpl32 intergenic spacer sequences Data type: species data This is an open access article distributed under the terms of the Creative Commons Attribution License (CC BY 4.0), which permits unrestricted use, distribution, and reproduction in any medium, provided the original author and source are credited.Joanne Pei-Chih Tan, Sheh May Tam, Ruth Kiew
