# Supplementary material for: Begoniayenyeniae (Begoniaceae), a new species from Endau Rompin National Park, Johor, Malaysia
Source: PhytoKeys. 2018 Nov 1;(110):23–37. doi: 10.3897/phytokeys.110.25846 (PMC6224366; doi:10.3897/phytokeys.110.25846)
Supplement: Supplementary material 1 — List of taxa with accession and location information for the 48 samples of Begonia included in the Bayesian analysis using ndhF-rpl32 intergenic spacer sequences [file phytokeys-110-023-s001.docx]

Supplementary file 1. List of taxa with accession and location information for the 48 samples of *Begonia* included in the Bayesian analysis using ndh*F*-rpl*32* intergenic spacer sequences

| **No** | **Species** | **Accession** | **Location of collection** |
| --- | --- | --- | --- |
|  |  |  |  |
| 1 | *B. wrayi* | FRI 80702 | Temenggor Dam, Perak |
| 2 | *B. wrayi* | FRI 75717 | Ulu Bertam, Gombak, Selangor |
| 3 | *B. wrayi* | FRI 63017 | G. Inas Forest Reserve, Baling, Kedah |
| 4 | *B. rhoephila* | FRI 75718 | Sg. Pisang, Selangor |
| 5 | *B. rheipholia* | NR1 | Rimba Ilmu, Universiti Malaya, Kuala Lumpur |
| 6 | *B. pavonina* | FRI 75725 | Cameron Highlands, Pahang |
| 7 | *B. sinuata* | FRI 75719 | Sg. Pisang, Selangor |
| 8 | *B. sinuata* (duplicate) | FRI 75719 | Sg. Pisang, Selangor |
| 9 | *B. longifolia* | FRI 75726 | Cameron Highlands, Pahang |
| 10 | *B. lowiana* (BN16) | CHRK 5442 | Cameron Highlands, Pahang |
| 11 | *B. decora* | FRI 75723 | Cameron Highlands, Pahang |
| 12 | *B. venusta* | FRI 75722 | Cameron Highlands, Pahang |
| 13 | *B. venusta x B. decora* | FRI 75724 | Cameron Highlands, Pahang |
| 14 | *B. rajah* | FRI 88682 | Cultivated |
| 15 | *B. yenyeniae* | FRI 47082 | Endau Rompin State Park, Segamat, Johor |
| 16 | *B. nurii* | FRI 75462 | Gua Gunting, Merapoh, Pahang |
| 17 | *B. nurii* | NR2 | Chiku 7, Gua Musang, Kelantan |
| 18 | *B. nurii* | NR3 | Gua Tabung, Merapoh, Pahang |
| 19 | *B. nurii* | FRI 82473 | Gua Gajah, Merapoh, Pahang |
| 20 | *B. nurii* | FRI 78436 | Gua Ulu Kumbang, Merapoh, Pahang |
| 21 | *B. kingiana* | FRI 78124 | Gunung Kanthan, Perak |
| 22 | *B. kingiana* | KBG 20090310 | Langkawi, Kedah |
| 23 | *B. rajah* X *B. kingiana* (artificial hybrid) | KBG 20180140 | FRIM nursery |
| 24 | *B. maxwelliana* | FRI 70306 | Bukit Larut, Perak |
| 25 | *B. harveyana* | FRI 49292 | Bukit Senggeh, Melaka |
| 26 | *B. sibthorpioides* | FRI 75877 | Gua Jerai, Kedah |
| 27 | *B. sibthorpioides* | FRI 75877 | Gua Jerai, Kedah |
| 28 | *B. sibthorpioides* | FRI 82520 | Gunung Machinchang, Langkawi, Kedah |
| 29 | *B. holttumii* | FRI 55986 | Cameron Highlands, Pahang |
| 30 | *B. barbellata* | NR4 | FRIM old nursery |
| 31 | *B. elisabethae* | KBG 20120162 | Langkawi, Kedah |
| 32 | *B. jiewhoei* | FRI 70623 | Gua Musang, Kelantan |
| 33 | *B. ignorata* | FRI 60008 | Gua Senyum, Pahang |
| 34 | *B. ignorata* | NR5 | FRIM old nursery |
| 35 | *B. foxworthyi* | NR6 | Chiku 7, Gua Musang, Kelantan |
| 36 | *B. foxworthyi* | FRI 68129 | Lentang Forest Reserve, Bentong, Pahang |
| 37 | *B. foxworthyi* | NR7 | Merapoh, Pahang |
| 38 | *B. foxworthyi* | NR8 | Chiku 8, Gua Musang, Kelantan |
| 39 | *B. jayaensis* | FRI 88520 | Gua Jaya, Kelantan |
| 40 | *B. jayaensis* | RK4912 | Gua Jaya, Kelantan |
| 41 | *B. kiamfeei* | S 95698 | Sarawak |
| 42 | *B. integrifolia* | KBG 20096677 | FRIM nursery (Ong’s orchid pot green form) |
| 43 | *B. speluncae* | SFC 3881 | Sarawak |
| 44 | *B. pubescens* | SFC 4211 | Sarawak |
| 45 | *B. sibthorpioides x B. sinuata* | FRI 80843 | Gunung Machinchang, Langkawi, Kedah |
| 46 | *B. tigrina* | FRI 66588 | Gua Setir, Kelantan |
| 47 | *B. abdullahpieei* | FRI 64721 | Sg. Lata Puteh, Bintang Hijau Forest Reserve, Larut, Perak |
| 48 | *B. reginula* | FRI 82471 | Ulu Senting, Negeri Sembilan |
